# Electrotonic Coupling in the Pituitary Supports the Hypothalamic-Pituitary-Gonadal Axis in a Sex Specific Manner

**DOI:** 10.3389/fnmol.2016.00065

**Published:** 2016-08-18

**Authors:** Christina Göngrich, Diego García-González, Corentin Le Magueresse, Lena C. Roth, Yasuhito Watanabe, Deborah J. Burks, Valery Grinevich, Hannah Monyer

**Affiliations:** ^1^Department of Clinical Neurobiology, Medical Faculty of Heidelberg, German Cancer Research Center, University of HeidelbergHeidelberg, Germany; ^2^Schaller Research Group on Neuropeptides, German Cancer Research Center, CellNetwork Cluster of Excellence, University of HeidelbergHeidelberg, Germany; ^3^Laboratory of Molecular Endocrinology, Centro de Investigación Príncipe FelipeValencia, Spain; ^4^Central Institute of Mental HealthMannheim, Germany

**Keywords:** gap junctions, hypothalamus, pituitary gland, reproduction, sex specificity

## Abstract

Gap junctions are present in many cell types throughout the animal kingdom and allow fast intercellular electrical and chemical communication between neighboring cells. Connexin-36 (Cx36), the major neuronal gap junction protein, synchronizes cellular activity in the brain, but also in other organs. Here we identify a sex-specific role for Cx36 within the hypothalamic-pituitary-gonadal (HPG) axis at the level of the anterior pituitary gland (AP). We show that Cx36 is expressed in gonadotropes of the AP sustaining their synchronous activity. Cx36 ablation affects the entire downstream HPG axis in females, but not in males. We demonstrate that Cx36-mediated coupling between gonadotropes in the AP supports gonadotropin-releasing hormone-induced secretion of luteinizing hormone. Furthermore, we provide evidence for negative feedback regulation of Cx36 expression in the AP by estradiol. We thus, conclude that hormonally-controlled plasticity of gap junction communication at the level of the AP constitutes an additional mechanism affecting female reproduction.

## Introduction

In vertebrates, gap junctions are formed by the connexin protein family, which consists of 20 isoforms in mice (Bennett and Zukin, 2004; Söhl et al., 2005). Connexin-36 (Cx36) is the major neuronal connexin isoform in rodents. In contrast to most other connexins, it forms exclusively homomeric and homotypic channels interconnecting selectively specific neuronal subtypes in many brain areas, including hippocampus, neocortex, reticular thalamus, olfactory bulb, and cerebellum (Al-Ubaidi et al., 2000; Belluardo et al., 2000; Deans et al., 2001; Hormuzdi et al., 2001; Long et al., 2002). In peripheral tissues Cx36 regulates insulin secretion from pancreatic ß-cells and catecholamine release from the adrenal medulla (Serre-Beinier et al., 2000; Martin et al., 2001; Ravier et al., 2005; Desarmenien et al., 2013). It is further expressed in the anterior pituitary (AP) gland, however, neither a function nor the cell type expressing Cx36 have been identified (Belluardo et al., 2000).

Episodic secretion of gonadotropin-releasing hormone (GnRH) from hypothalamic GnRH neurons elicits increases of [Ca^2+^]_i_ in gonadotropes leading to the pulsatile release of luteinizing hormone (LH) and follicle-stimulating hormone (FSH; Baird, 1978; Belchetz et al., 1978; Wildt et al., 1981; Tse et al., 1993). The mechanism of synchronous GnRH release from the median eminence has been much studied and includes both intrinsic and extrinsic factors. For instance, GnRH neurons are connected via the so-called “dendrons,” i.e., contacts serving both as dendrites and axons that support synchronous activity (Campbell et al., 2009). Of the external neuromodulators, kisspeptin neurons in the hypothalamic arcuate nucleus merit special mentioning (Yip et al., 2015). They are electrically coupled by Cx36-containing gap junctions (Campbell et al., 2011), and ablation of the kisspeptin gene prevents pulsatile LH release (Uenoyama et al., 2015). Conversely, optogenetic stimulation of kisspeptin neuron terminals induces pulsatile LH release in mice (Han et al., 2015).

Downstream of the pituitary, LH and FSH control the maturation of ovarian follicles, estradiol secretion, and ovulation in females (Wang and Greenwald, 1993). Whilst the pulsatile nature of gonadotropin secretion is controlled by GnRH, the frequency and amplitude are modulated by gonadal steroids (Belchetz et al., 1978; Leipheimer et al., 1984). However, it is not known whether additional local processes at the level of the AP itself may contribute to the fine-tuning and synchronization of gonadotrope activity that is necessary to generate the sharp LH and FSH pulses known to be present in the circulation. The presence of gap junctions in the AP between somatotropes, lactotropes, and folliculo-stellate cells has been evidenced by pharmacological and physiological studies (Guérineau et al., 1998; Fauquier et al., 2001; Hodson et al., 2012). A similar local mechanism for gonadotropes can be inferred from an *in vitro* study in which the authors demonstrated persistent rhythmic secretion of LH from an isolated pituitary (Gambacciani et al., 1987).

In this study we demonstrate the presence of gap junctions between gonadotropes, report reduced fertility in Cx36^−/−^ females, and show decreased LH secretion as a result of impaired gap junctional coupling between gonadotropes in the AP. Furthermore, we identify a negative feedback on Cx36 expression, exerted by ovary-derived estradiol.

## Materials and methods

All mice were housed at the standard animal facility in polypropylene macrolon cages with food and water *ad libitum* (12 h light-dark cycle 7 a.m.–7 p.m., temperature at 20 ± 1°C, humidity 50 ± 10%). Unless otherwise indicated, the mice were between 5 and 6 months old at the start of the experiments. All animal tests were approved by the ethics committee of the Regierungspräsidium Karlsruhe (Germany). The experimenter was unaware of the genotype.

### Fertility and puberty analyses

Eight to nine week old virgin females were mated with wt males for 10 days. For each mating pair the success of mating was scored, the time until the female littered down was measured and the number of pups per litter was counted. To determine puberty onset female mice were observed daily for vaginal opening starting at P22. Vaginal opening was further confirmed in the following days.

### Estrus cycle analysis and hormone measurements

Prior to hormone measurements, the cycle stage of 5 to 7 months old females was determined by analysis of cytological changes in the vaginal epithelium. Mice were handled for 2 weeks, and vaginal smears were taken daily at 3 ± 1 h after lights-on. The cells were stained according to Papanicolaou's protocol (Papanicolaou, 1942) and cell populations were counted using a light microscope. Two to six consecutive estrous cycles were analyzed per female. For estradiol measurement serum was prepared from trunk blood of wt and Cx36^−/−^ female mice that were sacrificed in the afternoon of the first day of estrus. Estradiol concentration was measured by HPLC.

#### Plasma LH measurements

Six to seven months old male mice and female mice at the stage of estrus in the morning were sacrificed with CO_2_, and the blood was taken from the *vena cava inferior* into EDTA-containing syringes. The blood was collected on ice in EDTA-coated tubes (BD Mictrotainer™, Becton, Dickinson & Co, USA) and centrifuged 20 min at 4°C, 2000 rpm. Plasma was stored in Eppendorf tubes at −80°C. Mouse LH radioimmunoassay (RIA) was performed by the Endocrine Technology and Support Lab, Oregon National Primate Research Center (Beaverton, OR) using a traditional double-antibody RIA procedure similar to that described previously (Pau et al., 1986). The LH RIA kit was purchased from Dr. Albert Parlow (NHPP, Harbor-UCLA Medical Center, Los Angeles). The detection limit of the assay was 0.01953 ng/tube (i.e., 0.2 ng/ml). A mouse serum pool was used in triplicate in each assay as quality control. The interassay variation is estimated at 14.7% (*n* = 5 assays) and the intra-assay variation in those 5 assays averaged 3.83% (0.4–7.8%).

#### Plasma testosterone (T) measurements

Male mice were sacrificed with CO_2_ and samples (2 μl) were extracted in 5 ml ether in 13 X 100 glass tubes (baked at 500°C for 30 min), dried under forced air, and analyzed by specific T RIA. Hormonal values were corrected for extraction losses determined by radioactive trace recovery at the same time with sample extraction; hot recovery usually is better than 90%. The sensitivity was 5 pg/tube for the T RIA. The intra-assay variation was 5.1% for the T assay. Experiments were performed at the Endocrine Technology and Support Core Lab (ETSL) at the Oregon National Primate Research Center/Oregon Health & Science University (Rasmussen et al., 1984).

#### Haematoxylin-eosin staining of ovaries

After embedding in paraffin, serial 5 μm sections were prepared for H&E staining and quantification. Sections were deparaffinized and stained as described previously (Burks et al., 2000). Area and diameter measurements were carried out with ImageJ software (NIH). Representative images were obtained from 20 μm cryostat sections.

Estradiol measurements and analysis of ovarian morphology was statistically analyzed using Sigma Plot. For the remaining experiments the SPSS 16.0 software was used.

### Tissue preparation for histochemistry

Mice were deeply anesthetized by intraperitoneal injection of ketamine/xylazine, transcardially perfused, and the tissue was post-fixed with 4% parafomaldehyde (PFA) in PBS (pH 7.4). Pituitaries and brains were sectioned coronally using a Leica VT1000S vibratome (Leica, Germany). Ovaries were dissected from wt and Cx36^−/−^ females in the afternoon of the first day of estrus and fixed in 4% PFA.

### Immunohistochemistry

Free-floating sections were permeabilized with 0.2% TritonX-100 and blocked in 5% BSA prior to antibody incubation. Primary antibody incubation was carried out for 18–48 h at 4°C, secondary antibody incubation was carried out at room temperature (RT) for 2 h. Antibodies were chicken anti-EGFP (1:500, Abcam, ab13970), rabbit anti-LHRH (LR1; 1:3000, gift from Dr. R. Benoit, McGill University, Montreal, Canada), rabbit anti mouse-LH (1:2000, mybiosource.com, MBS220981), rabbit anti-mouse FSH (1:1000, Dr. A.F. Parlow, National Hormone and Pituitary Program, NIH) Secondary antibodies: anti-rabbit Alexa 488, anti-rabbit Alexa 555, anti-chicken Alexa 488 (1:1000, Invitrogen), anti-rabbit Cy3 (1:2000, Dianova), anti-chicken FITC, and anti-rabbit Cy3 (1:500; Jackson ImmunoResearch Laboratories, Inc.). Cells were counted using the cell counter plug-in in NIH ImageJ v1.48a.

### *In situ* hybridization combined with immunohistochemistry

Five months old female wt and Cx36^−/−^ mice were deeply anesthetized, and transcardially perfused with PBS for 2 min, followed by 4% PFA for 30 min, 5% glycerol with 0.5% Dimethylsulfoxid (DMSO) in PBS for 5 min, and 20% glycerol with 2% DMSO in PBS for 30 min. To minimize RNA degradation, protectRNA (Sigma-Aldrich) was added to 4% PFA and glycerol. Pituitaries were removed, frozen on dry ice, and kept at −80°C until use. Eighteen micrometer cryosections of the pituitary were prepared and attached to the same microscope slides. Sections were dried at room temperature (RT) for 30 min before performing *in situ* hybridization using a viewRNA ISH Tissue 2-Plex Assay kit and the viewRNA Type1 probe against mouse Cx36 (#VB1-18818, Affymetrix, UK). *In situ* hybridization was performed following the protocol of the manufacturer. In brief, sections were treated with protease for 10 min at 40°C, hybridized with the probe, and the probe was colorized. After *in situ* hybridization, sections were treated with a blocking solution (1% BSA and 0.2% TritonX-100 in PBS) for 1 h, and incubated with rabbit anti-rat LH beta (1:400, #AFP571292393, Dr. A. F. Parlow, National Hormone & Peptide Program, California) diluted in 0.2% TritonX-100 in PBS overnight at 4°C. Sections were washed with PBS and incubated with secondary antibody (1:500, donkey anti-rabbit IgG, Alexa Fluor 647 conjugate, A-31573, Thermo Fisher Scientific) for 2 h at RT. After washing with PBS, sections were counterstained with DAPI (1:500) diluted in water for 30 min at RT. Sections were washed again with PBS, air-dried for 20 min at RT and coverslipped using Fluoromount-G (00-4958-02, eBioscience, Germany).

### Electrophysiology

Pituitary sections were prepared from juvenile Cx36-EGFP mice (3–7 weeks). Mice were deeply anesthetized and killed by decapitation. The pituitary gland was removed and embedded in 2% Agar. Two-hundred and fifty micrometers thick coronal slices were cut using a vibratome (HR2, Sigmann Elektronik) in ice-cold ACSF containing (in mM): 120 NaCl, 3.5 KCl, 2.5 CaCl_2_, 1.3 MgSO4, 1.25 NaH_2_PO_4_, 25 NaHCO_3_, 25 glucose (pH 7.2). Whole-cell current clamp recordings were made at 30–32°C from visually identified EGFP-positive cells using an EPC8 amplifier (HEKA) and a confocal upright microscope (TCS SP5, Leica) equipped with IR-DIC and standard epifluorescence. Patch pipettes had a resistance of 4–6 MΩ when filled with (in mM): 105 Kgluconate, 30 KCl, 4 Mg-ATP, 10 phosphocreatine, 0.3 GTP, and 10 HEPES, adjusted to pH 7.3 with KOH (final osmolarity ~290 mOsm). Sulforhodamine (final concentration 20 μM) was routinely added to the intracellular solution. Cells were held at −60 mV. Signals were acquired using two EPC8 amplifiers and PatchMaster software (HEKA), sampled at 20 kHz, filtered at 3 kHz, and analyzed using IgorPro (Wavemetrics). The coupling coefficient was calculated as the ratio of the voltage response in cell 2 divided by the voltage response in cell 1 under steady-state conditions. It was obtained by averaging 10 to 60 consecutive sweeps. Data are presented as mean ± standard error of the mean (SEM).

### Calcium imaging

To rule out possible variations due to fluctuating expression levels of GnRH-R, Cx36, and hormones during the estrous cycle, mice were staged based on the cytological examination of the vaginal smear each day at the same time and 8–12 week old female wild-type and Cx36^−/−^ mice were sacrificed when they were in estrus. After decapitation under isofluorane anesthesia, the pituitary gland was carefully dissected out and embedded in 2% agarose (Applichem) in ACSF at 37°C. The slicing chamber was filled with ACSF and coronal slices (150 μm thick) were obtained using a vibratome (HR2, Sigmann Elektronik). Slices were immediately transferred to an incubator plate with ACSF under continuous bubbling at RT. Subsequently, slices were incubated in a solution containing the cell-permeable fluorescent Ca^2+^ indicator Fluo-4 AM (5 μM; Invitrogen), Cell Tracker Red CMTPX (10 μM; Invitrogen) to delineate cell membranes and pluronic F127 (0.02%; Invitrogen). Dye loading was carried out at RT for 40–45 min.

Imaging of fluorescently labeled cells in acute slices was performed on a TCS SP5 microscope (Leica) equipped with a 20x (1 numerical aperture) water-immersion objective. Images (512 × 512 pixels) were acquired at 1000 Hz speed every 0.5–2 s with 0.5 μm per pixel resolution in the xy dimension, and 4–5 μm steps in the z dimension. Argon and HeNe-543 lasers were used to excite Fluo-4 AM and Cell Tracker Red CMTPX dyes, respectively. After recording baseline fluorescence for 2 min, GnRH was applied (1 nM in ACSF; Sigma-Aldrich) for 2 min. Recordings continued for up to 26 min. ACSF was applied by a pump perfusion system with a constant flux (1.5 ml/min) that continuously renewed the buffer in the recording chamber. Long-lasting effects induced by GnRH were never observed, hence for practical reasons, for plots and statistical analyses only the first 10 min of recordings were considered. Leica Application Suite AF software was used to record and measure fluorescent activity in 7 independent experiments per group.

We obtained relative fluorescence changes (*F*_i_/*F*_0_), where *F*_0_ is the fluorescence image formed by averaging the first 50 frames of the sequence, and *F*_(i)_ represents each (i) frame of the recording. We then performed a large-scale analysis investigating rank correlation (Spearman Rank Order test) between ΔF values from all GnRH-responding cell pairs to test whether events of gross synchrony between cells occurred during the whole recording, as described previously (Bonnefont et al., 2005). We also studied the correlation coefficients of connected cell pairs along time windows (*p* < 0.001) and in consequence, we decided only to consider 3 meaningful time windows: before (between 100 and 200 s), during (between 250 and 350 s), and after (500–600 s) GnRH application. We performed complementary statistical tests to assess differences in correlation coefficients within each group (Mann–Whitney rank sum test). Distances between each GnRH-responding cell pair were measured (ImageJ, NIH). We first performed correlation analysis between correlation coefficients from GnRH-responding cells and intercellular distances. If there was a significant correlation, we subsequently compared slopes and intercepts of the regression lines. For this latter analysis, values of *p* < 0.001 were considered statistically significant.

Data are presented as mean ± SEM or as median ± IQR, as indicated. Statistical analyses were performed using Sigmaplot 11.0 or GraphPad Prism 5. To test for normal distribution of the data, the Kolmogorov–Smirnov test and equal variance by Levene median test were used. Differences between groups were examined using Student's *t*-test, one-way ANOVA with Bonferroni *t*-test for multiple-comparisons, or Mann–Whitney rank sum test as indicated. Values of *p* < 0.05 were considered statistically significant for the rest of the analyses.

### Gonadectomy

A small incision directly above the scrotum and, respectively cephally to the iliac crest was made under a mixture of ketamine hydrochloride (90 mg/kg) and xylazine (5 mg/kg) anesthesia dissolved in phosphate buffered saline (PBS). Testes or ovaries were extracted, ligated with a monofilament suture and removed. Muscle layers and skin were sutured, and mice were singly housed until full recovery from the anesthesia. Blood and tissues were collected 1 week after surgery.

### Pituitary cell culture

Eight to sixteen week old wild-type females were used. After decapitation under isoflurane anesthesia, the pituitary gland was dissected out in ice cold Hanks balanced salt solution (HBSS; Life Technologies) plus 10 mM HEPES. Following 45 min incubation at 37°C in 0.25% trypsin, cells were triturated in the presence of 0.05% DNAseI. Trypsin was blocked with 10% FBS. Cells were washed twice with DMEM (Life Technologies) without phenol red and plated in a poly-lysine (10 ug/ml in borate buffer, pH 8.4) coated 24 well plate at 1.5 × 10^5^ cells per well. DMEM containing FBS (5%; Life Technologies), Pen/Strep (1%; Life Technologies), non-essential amino acids (1%; Life Technologies), sodium pyruvate (1%; Life Technologies), and Glutamax (1%; Life Technologies) was used as culture medium. After 2 days *in vitro* (DIV), the cells underwent a 12 h serum starvation and were then stimulated with 17ßestradiol (100 nM; Sigma-Aldrich) for 3 or 6 h.

### qPCR analysis

Tissues were rapidly dissected, frozen on dry ice and stored at −80°C. Cells were lysed in RLT buffer and stored at −80°C. Total RNA was extracted using the RNeasy Mini Kit (Qiagen, Hilden, Germany). After DNaseI digestion, cDNA was synthesized using random hexamers and SuperScript II reverse transcriptase (Life Technologies, Darmstadt, Germany). Quantitative PCR analysis was performed using the StepOnePlus continuous fluorescence detector (Applied Biosystems Research, Foster City, CA, USA). Product amplification was determined by SYBR Green 1 fluorescence detection. Standard cycling procedures were employed with annealing temperatures of 60°C. Specific amplicon formation with each primer pair was confirmed by melt curve analysis. Gene expression was quantified relative to 18S ribosomal RNA. qPCR measurements were performed in duplicate or triplicate. Primers were: Cx36 sense: 5′ TGGCTTCAGTGTTCCA GGCTTGTA 3′, Cx36 antisense: 5′ CGCTCACAGCAAACATGAACACCA 3′, 18S sense: 5′ CACACGCTGAGCCAG 3′, 18S antisense: 5′ AGGTTTGTGATGCCC 3′.

### Promoter analysis

Position weight matrices for ERα and ERß were retrieved using the MotifDB (Shannon, 2014) package for R (R Core Team, 2015) and matched to the Cx36 promoter retrieved from the UCSC mm10 genome following the workflow “Gene Regulation Transcription Factor Binding Sites” published on bioconductor (http://www.bioconductor.org/).

## Results

**Figure 1:**
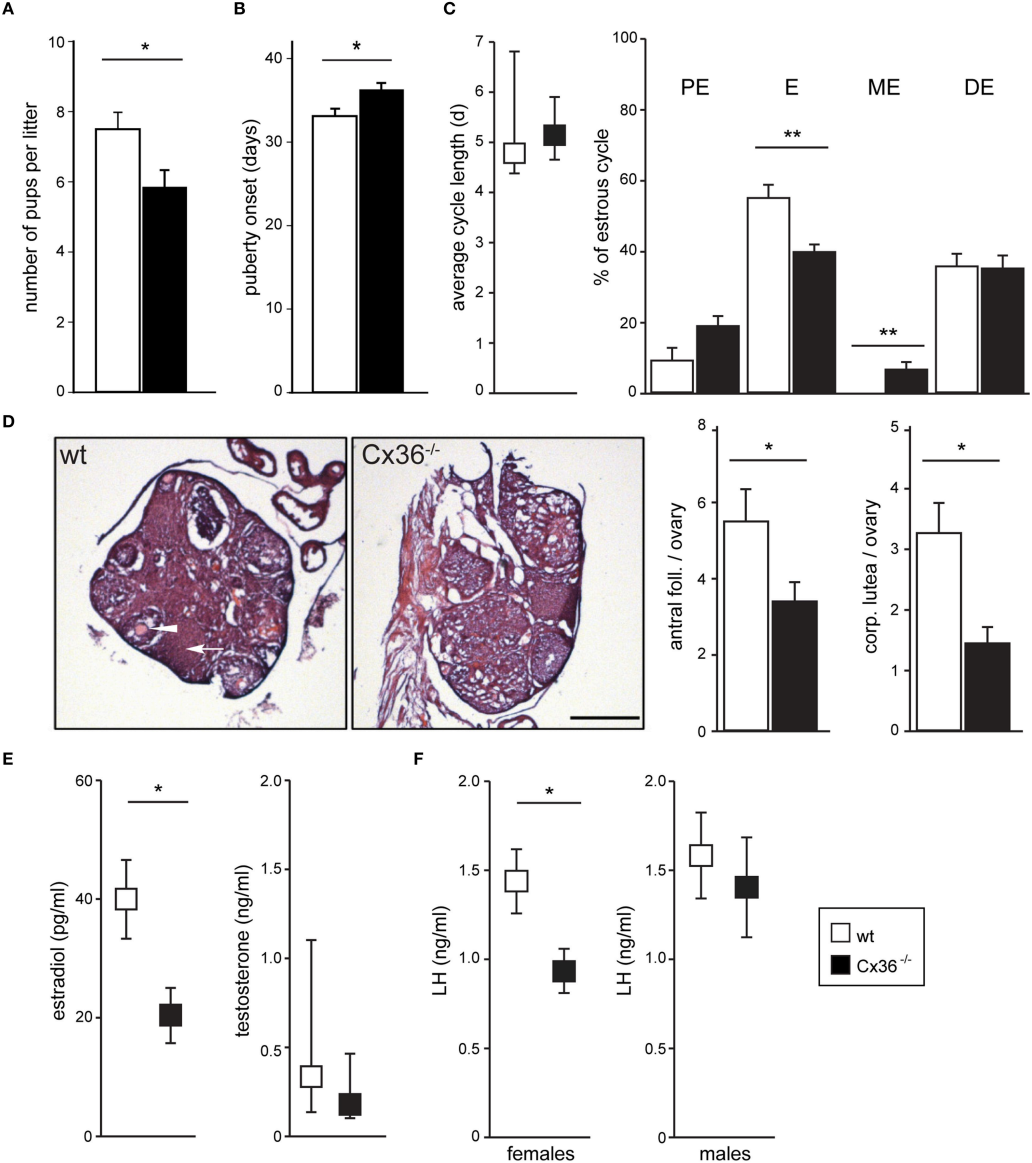
**Cx36 ablation affects the HPG axis downstream of the pituitary gland in a sex-specific fashion**. **(A)** Cx36^−/−^ females produce smaller litters than wt females when mated with wt males. Wt females: *n* = 12 mice; Cx36^−/−^ females: *n* = 13 mice; Mean ± SEM; ^*^P ≤ 0.05, Student's *t*-test. **(B)** Puberty onset is delayed in Cx36^−/−^ females. Wt females: *n* = 17 mice; Cx36^−/−^ females: *n* = 15 mice; Mean ± SEM; ^*^P ≤ 0.05, Student's *t*-test. **(C)** Cx36 ablation differentially affects estrous cycle phases. The total duration of the estrous cycle was comparable in wt and Cx36^−/−^ females (left panel, *n* = 5 mice/genotype; median [IQR], Mann–Whitney *U*-test). However, while estrus duration was 55.04 ± 3.6% of the total cycle length in wt mice, it was reduced to 39.7 ± 1.98% in Cx36^−/−^ mice. Conversely, metestrus was on average 6.42 ± 1.9% of the total cycle time in Cx36^−/−^ mice, while this stage was too short to be detected in wt mice. Mean ± SEM. ^**^P ≤ 0.01, Student's *t*-test, PE, proestrus; E, estrus; ME, metestrus; DE, diestrus. **(D)** Ovaries in Cx36^−/−^ mice exhibit histological alterations. Representative images of hematoxylin and eosin stained sections from wt and Cx36^−/−^ ovaries. Averaged number of antral follicles and of *corpora lutea* per ovary at the first day of estrus in wt (*n* = 10 ovaries/5 mice), Cx36^−/−^ (*n* = 12 ovaries/6 mice). Scale bar: 500 μm; mean ± SEM. ^*^P ≤ 0.05; ^**^P ≤ 0.01; Student's *t*-test. **(E)** Following Cx36 ablation, plasma estradiol concentration (measured by HPLC on first day of estrus) is decreased in females whereas testosterone levels in males (measured by RIA) are not affected. Wt females: *n* = 6 mice; Cx36^−/−^ females: *n* = 7 mice. Mean ± SEM; ^*^P ≤ 0.05, Student's *t*-test. *n* = 8 male mice/genotype. Median [IQR], Mann–Whitney *U*-test. **(F)** Following Cx36 ablation, plasma LH concentration (determined by RIA) is altered in females (measured on the first day of estrus), but not in males. Wt females: *n* = 14 mice; Cx36^−/−^ females: *n* = 11 mice; *n* = 8 male mice/genotype. Mean ± SEM; ^*^P ≤ 0.05, Student's *t*-test.

To evaluate the input of Cx36 on female reproduction, we performed fecundity tests by mating wild-type (wt) or Cx36^−/−^ (Hormuzdi et al., 2001) virgin females with wt proven stud males, and found that Cx36^−/−^ females produced smaller litters than control females (Figure 1A), while both the percentage of successful matings and the time to produce the first litter were unaltered (data not shown). Furthermore, puberty onset measured as day of vaginal opening was delayed by 3 days in Cx36^−/−^ females (Figure 1B). Prompted by these findings we continued to characterize the reproductive phenotype in more detail. Analysis of the estrous cycle based on vaginal smear cytology showed that the total duration was unchanged in Cx36^−/−^ compared to wt females (Figure 1C). However, the relative time spent in estrus was significantly reduced, while the duration of metestrus was prolonged in Cx36^−/−^ mice (Figure 1C). Histological analysis of ovaries harvested on the first day of estrus showed a drastic reduction in the number of antral follicles and *corpora lutea* in Cx36^−/−^ mice compared to control (Figure 1D), which is suggestive of reduced ovulation. Accordingly, plasma concentrations of estradiol were decreased by 47% in Cx36^−/−^ females, and LH concentrations were reduced by 40%, when both were measured in the morning on the first day of estrus (Figures 1E,F). Of note, plasma testosterone levels as well as plasma LH in males were unchanged (Figures 1E,F), suggesting a specific role of Cx36 in the female hypothalamic-pituitary-gonadal (HPG) axis.

Intrigued by these observations we set out to investigate the role of Cx36 within the HPG axis, and first established the site and pattern of expression. We quantified Cx36 mRNA expression levels in pituitaries and gonads from male and female wt mice using qPCR. The expression level in the pituitary gland was comparable in the two sexes, and there was only very low or no expression in testes and ovaries, respectively (Supplementary Figure 1A). Given the heterogeneity of the hypothalamus with respect to function, we investigated Cx36 expression directly in GnRH neurons. To this end we employed transgenic mice that we had previously characterized (Christie et al., 2005) and that showed faithful expression of the fusion protein Cx36-EGFP under the control of the Cx36 promoter. In agreement with a previous study, (Campbell et al., 2011) we found no evidence for Cx36-EGFP expression and therefore Cx36 mediated gap junction coupling between GnRH neurons (Supplementary Figure 1B). Thus, within the HPG axis, we detected Cx36 expression only in the pituitary gland.

**Figure 2:**
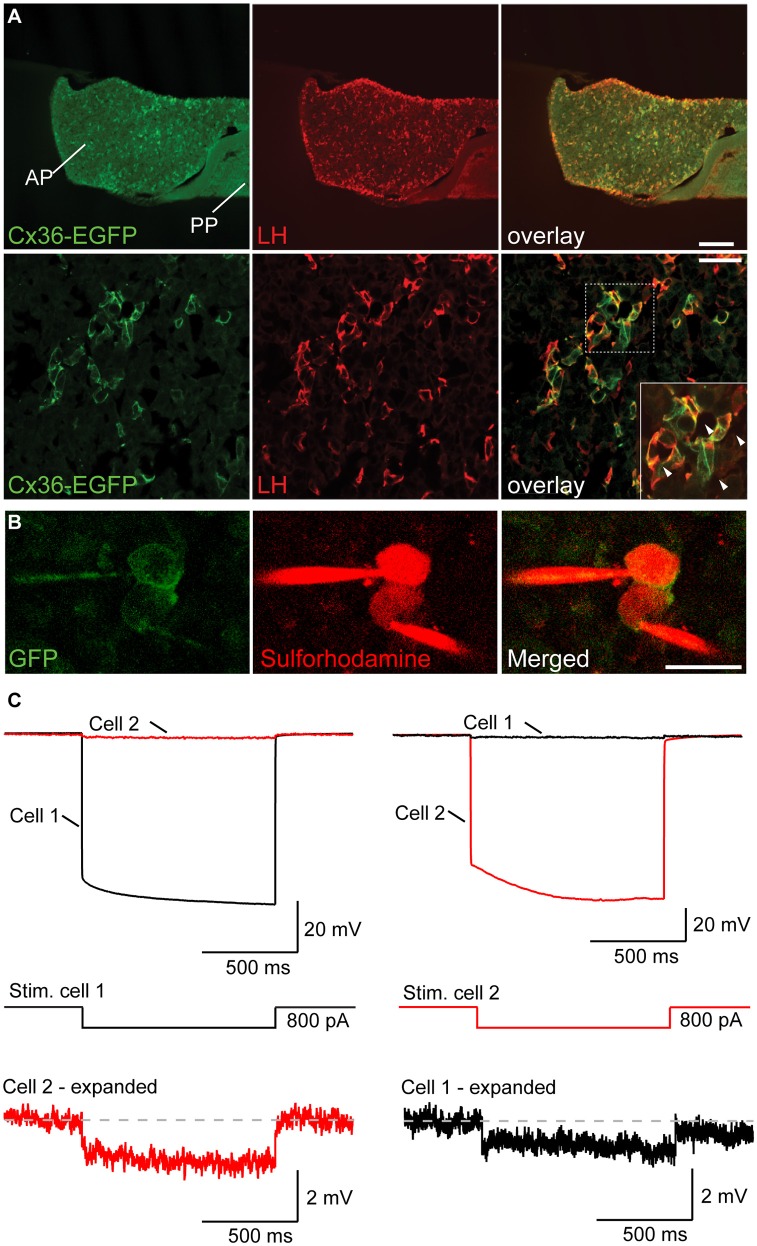
**Gonadotropes of the pituitary gland are coupled via Cx36-containing gap junctions. (A)** Cx36-EGFP is expressed in LH^+^ cells in the AP. Lower panels show higher magnification. Arrowheads in the inset highlight the localization of Cx36-EGFP signal to membrane appositions of LH^+^ cells. AP, anterior pituitary; PP, posterior pituitary. **(B)** Confocal microscopy images of two adjacent EGFP^+^ cells in the AP immediately before (left panels) and after (right panels) whole-cell patch-clamp. Sulforhodamine (in red) was added to the intracellular solution to help visualize patch-clamped cells. **(C)** The voltage response of cell 1 (top left) after hyperpolarizing current injection (middle) is detectable in cell 2, albeit with a much reduced amplitude. Conversely, the voltage response of cell 2 (top right) can be detected in cell 1. Six out of nine tested pairs showed such a response.

To determine the cellular identity of Cx36 expressing cells in the anterior pituitary (AP), we took again recourse to Cx36-EGFP transgenic female mice. Double labeling for EGFP and LH (as well as EGFP and FSH) revealed that 99.63 ± 0.37% of LH^+^ cells (as well as 87.27 ± 2.63% of FSH^+^ cells) expressed EGFP. Conversely, the percentage of EGFP^+^ cells expressing either LH or FSH was 99.62 ± 0.37%, and 95.00 ± 2.26%, respectively (mean ± SEM, *n* = 3 mice), showing that Cx36 was almost exclusively expressed by gonadotropes of the AP (Figure 2A and Supplementary Figure 1C). In control experiments we established that Cx36 ablation did not affect the average number of gonadotropes (Supplementary Figure 2). Higher magnification images revealed that Cx36-EGFP localized frequently to membrane appositions of LH^+^ cells (Figure 2A, arrowheads), suggesting that gonadotropes might be connected via Cx36-containing gap junctions. We further confirmed the specific expression of Cx36 in gonadotrope cells in the pituitary of wild-type female mice employing *in situ* hybridization to detect Cx36 mRNA, and immunohistochemistry against LH (Supplementary Figures 1D,E).

To provide functional evidence for gap junction mediated coupling between gonadotropes, we performed paired patch-clamp recordings from Cx36-EGFP expressing gonadotropes in acute slices of young female mice (Figure 2B). As previously shown, we can exclude that the Cx36-EGFP fusion protein forms channels on its own as it needs to partner with wt Cx36 (Helbig et al., 2010). In 6 out of 9 tested Cx36-EGFP positive cell pairs, hyperpolarizing current injection in one cell elicited a detectable voltage deflection in the other cell (Figure 2C). On average, electrical coupling was symmetrical, i.e., was not dependent on which cell of the pair underwent current injection, thus demonstrating the presence of gap junctions between gonadotropes. The strength of coupling was 0.025 ± 0.008 (*n* = 6).

**Figure 3:**
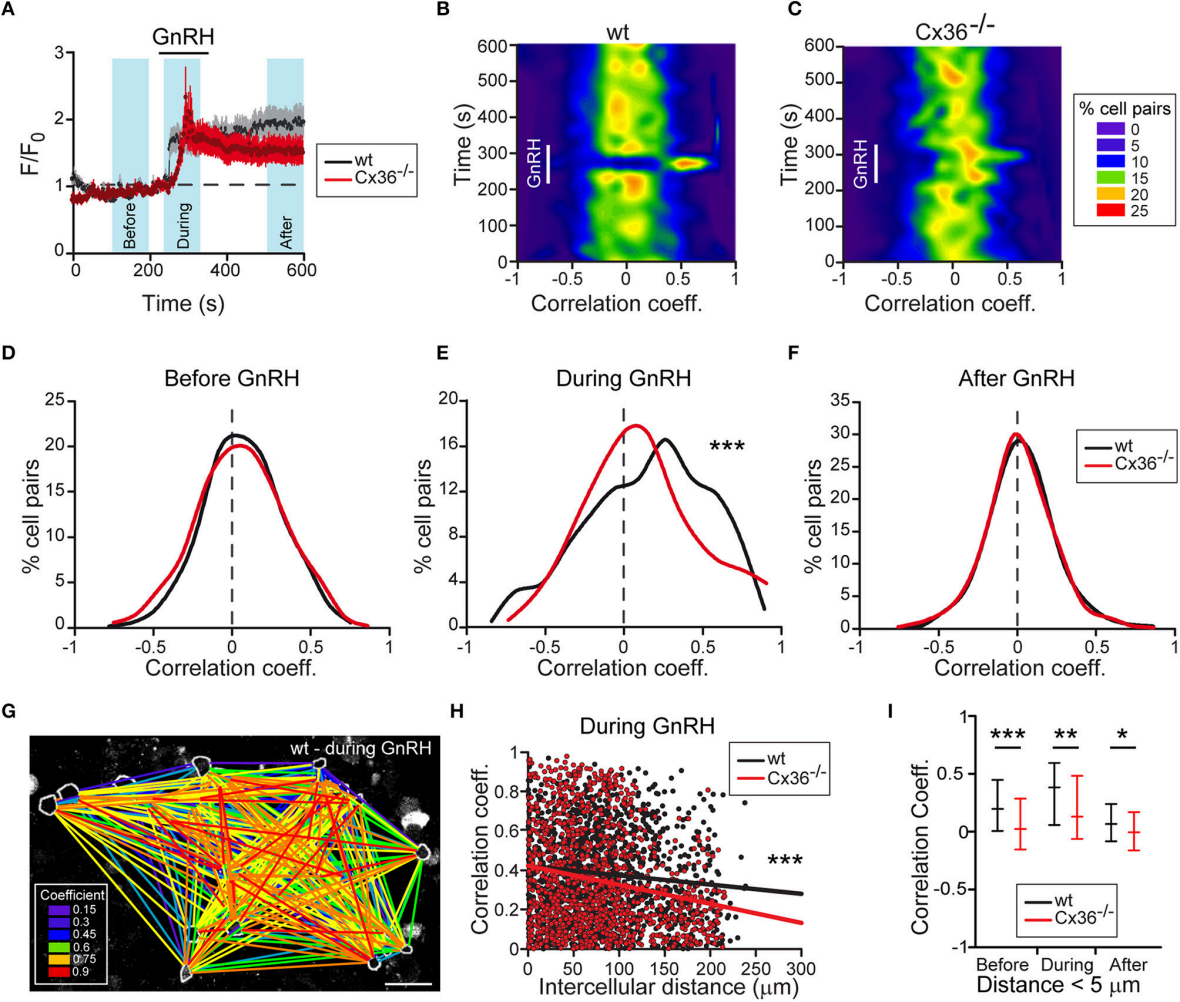
**Deficient electrical coupling between gonadotropes in Cx36^−/−^ mice as revealed by Ca^2+^ imaging. (A)** GnRH-induced calcium response in wt and Cx36^−/−^ AP. Representative examples of averaged recordings from one experiment. GnRH (1 nM) was applied for 2 min. Number of cells: wt *n* = 32; Cx36^−/−^ *n* = 14. Data represent mean ± SEM. Blue areas represent time windows selected for statistical analysis. **(B,C)** GnRH evokes stronger and more coordinated responses in wt **(B)** than in Cx36^−/−^ **(C)** pituitaries. Representative heat maps from one wt and one Cx36^−/−^ AP (528 and 351 cell pairs, respectively) showing temporal dynamic changes of correlated activity (Spearman coefficient). **(D–F)** Cx36 ablation effects GnRH-induced, but not basal synchrony. Distribution of Spearman correlation coefficients for cell pairs before **(D)**, during **(E)**, and after **(F)** GnRH application. Although some cell pairs displayed high coefficient correlation values, no large-scale synchrony was observed before GnRH application. Seven experiments/genotype; wt *n* = 1908; Cx36^−/−^ *n* = 1951 cell pairs, Mann–Whitney Rank Sum Test. ^***^P ≤ 0.001. **(G)** Illustrative example of one correlation map identifying GnRH-responsive cell pairs in wt mice during GnRH application. All GnRH-responsive cell pairs are connected with lines. Colors represent the correlation intensity according to Spearman correlation coefficients. Scale bar: 25 μm. **(H)** Comparative linear regression analysis between the positive values of Spearman correlation coefficients and intercellular distances showing that only during GnRH application the slopes are significantly different when comparing wt and Cx36^−/−^. For more details, see Supplementary Figure S3. Seven experiments/genotype; wt *n* = 1908; Cx36^−/−^ *n* = 1951 cell pairs. ^***^P ≤ 0.001. **(I)** Synchrony between Cx36^−/−^ cells is decreased also before and after GnRH application when considering only short-distance coupling (< 5 μm). Spearman correlation coefficients obtained from 7 experiments/genotype, wt *n* = 82; Cx36^−/−^ *n* = 93 cell pairs, median [IQR], Mann-Whitney Rank Sum Test. ^*^P ≤ 0.05, ^**^P ≤ 0.01; ^***^P ≤ 0.001.

To substantiate the functional role of Cx36 on the activity of gonadotropes, we assessed GnRH-induced Ca^2+^ responses in acute pituitary slices from wt and Cx36^−/−^ mice that were in estrus. We detected GnRH-induced increases in intracellular Ca^2+^ both in wt and Cx36^−/−^ pituitary cells (Figure 3A). In both genotypes, typically two types of responses were observed, namely biphasic or oscillatory responses (Supplementary Figures 3A–D). The degree of synchrony was determined by performing a pairwise comparison of the relative fluorescence values for all GnRH responsive cells and averages of one experiment were plotted as heat maps (Figures 3B,C). Notably, there was an increase in the correlation coefficients in the wt upon GnRH stimulation. There was no difference before and after GnRH stimulation between genotypes (Figures 3D,F), whilst GnRH stimulation led to an increase in the number of cell pairs with enhanced synchrony in wt compared to Cx36^−/−^ (Figure 3E). We further evaluated the influence of Cx36 deletion on the network activity by taking into consideration the intercellular distance for each cell pair (Figure 3G). Indeed, at a larger scale, Cx36 ablation affected the synchrony of network activity only during the application of GnRH (Figure 3H and Supplementary Figure 4). Thus, there was a steeper negative slope of the regression line for correlation coefficients between cell pairs obtained from Cx36^−/−^ pituitary slices. This reflects decreased coupling with increasing intercellular distance, thus highlighting the role of gap junction coupling in the entrainment of gonadotropes to a common activity pattern. Surprisingly, average correlation coefficients between cells in close proximity (up to 5 μm distance between cell membranes) were reduced in Cx36^−/−^ slices throughout the experiment, unmasking a role for gap junction coupling under both basal and stimulated conditions (Figure 3I).

**Figure 4:**
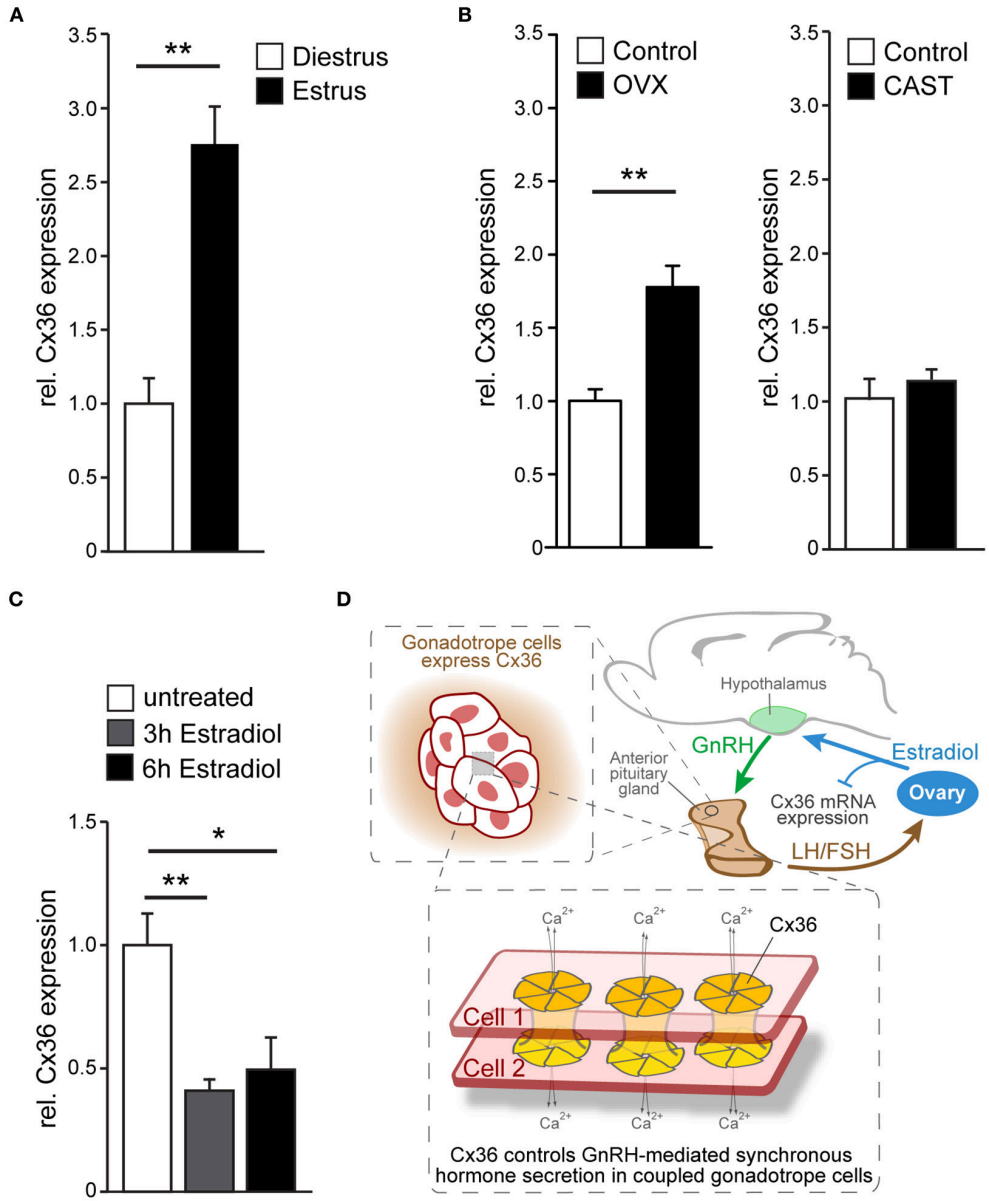
**Cx36 expression is regulated in a sex-specific manner. (A)** Cx36 mRNA expression in the pituitary gland of female wt mice is augmented in estrus (*n* = 9) compared to diestrus (*n* = 5) as shown by qPCR analysis. Mean ± SEM; ^**^P ≤ 0.01; Student's *t*-test. **(B)** Ovariectomy results in increased Cx36 mRNA expression (*n* = 6, control *n* = 9) while castration (*n* = 4, control *n* = 3) has no effect on Cx36 mRNA expression in the pituitary gland of males. Mean ± SEM; ^**^P ≤ 0.01; Student's *t*-test. **(C)** Treatment of cultured pituitary cells from wt females with estradiol leads to a rapid suppression of mRNA expression. *n* = 4 independent experiments, performed in triplicate or quadruplicate. Mean ± SEM; ^**^P ≤ 0.01; ^*^P ≤ 0.05; ANOVA, Bonferroni *post-hoc* test. **(D)** Schematic diagram illustrating Cx36 action on the hypothalamic-pituitary-ovarian axis. Cx36 controls GnRH-mediated synchronous hormone secretion (LH/FSH) in coupled gonadotrope cells in the pituitary gland. LH and/or FSH stimulate estradiol production in the ovaries, which in turn represses Cx36 mRNA expression in the pituitary gland.

Given the estrous cycle dependent pattern of LH secretion, we wondered whether Cx36 expression itself is regulated across the estrous cycle. Hence, we analyzed Cx36 mRNA in estrus and diestrus, and found a two-fold increase of Cx36 mRNA in estrus (Figure 4A). Subsequently, we examined Cx36 expression following ovariectomy and orchiectomy to investigate whether sex steroids played a role in the regulation of Cx36 expression. Of note, ovariectomy in females caused an about two-fold increase of Cx36 expression, while castration of males had no impact on Cx36 expression levels (Figure 4B). Using a bioinformatics approach, we searched 5 kb of the Cx36 promoter sequence upstream of the transcription start site (TSS) for putative estrogen response elements to determine if the repression of Cx36 expression could be a direct effect of ER binding to the promoter region of Cx36. In support of this hypothesis, we found two putative binding sites for ERalpha with 75% minimal matching scores located 725 and 3109 bp upstream of the TSS (Supplementary Figure 5A), and one putative binding site for ERbeta 2458 bp upstream of the TSS with an 88% minimal matching score (Supplementary Figure 5B). To address if estradiol can directly influence the expression level of Cx36 in gonadotropes, we exposed cultured wt pituitary cells to estradiol for 3 or 6 h. Indeed, this treatment induced a significant decrease of Cx36 mRNA expression compared to controls, further corroborating the finding that steroid hormones, specifically estradiol, regulate Cx36 expression (Figure 4C).

Thus, suppression of Cx36 mRNA by ovarian steroids is indicative of a gender-specific gap junction function within the HPG axis, specifically in the control of GnRH-mediated synchronous gonadotrope secretion.

## Discussion

In summary our results demonstrate that Cx36 mediated cell-cell communication within the AP is essential to translate pulsatile GnRH stimulation into appropriate simultaneous activation of gonadotropes in females, and that this cell-cell communication is negatively regulated by estradiol (Figure 4D). In the absence of Cx36, LH levels were decreased, estrous cycle phases were shifted, ovulation was impaired, and litter size was reduced. We postulate that the effects within the HPG axis in female mice are downstream of GnRH neurons since they were not observed in mutant mice in which Cx36 was conditionally ablated in forebrain neurons (Campbell et al., 2011).

Gap junctions have been reported to couple other types of pituitary cells where they help to maintain basal activity and mediate synchronous population responses upon stimulation (Guérineau et al., 1998; Fauquier et al., 2001; Hodson et al., 2012). Here we directly demonstrate the presence of gap junctions between gonadotropes in the AP by simultaneous electrophysiological recordings from pairs of neighboring gonadotropes. After establishing the exclusive expression of Cx36-EGFP in gonadotropes, we took advantage of this finding to identify gonadotropes in live pituitary slices. Paired patch-clamp recordings of neighboring EGFP-positive cells revealed symmetrical electrical coupling, thus demonstrating the presence of gap junctions (Hormuzdi et al., 2001). The coupling coefficient was in the lower range of what was reported previously for neurons coupled via Cx36-containing gap junctions (Gibson et al., 1999; Meyer et al., 2002). Gap junctions between gonadotropes appear to be a common feature of this cell type as they were detected between 67% of tested cell pairs. This is similar to the extent of gap junction coupling identified by dye-transfer between lactotropes and somatotropes (Guérineau et al., 1998; Hodson et al., 2012).

Using Ca^2+^ imaging experiments in acute pituitary slices from wt and Cx36^−/−^ females we established the functional relevance of this coupling at the level of the gonadotrope network. Ablation of Cx36 resulted in asynchronous Ca^2+^ activity in gonadotropes upon GnRH stimulation. Taking into account only cells in close proximity, i.e., neighboring cells, we found a significant decorrelation of Ca^2+^ activity also under basal conditions in Cx36 deficient pituitary slices. Similarly, Ca^2+^ transients in spontaneously coactive somatotropes were reported to desynchronize under basal conditions upon incubation with the general gap junction blocker halothane (Guérineau et al., 1998). Our findings show that Cx36 is crucial to maintain synchrony of Ca^2+^ transients between neighboring gonadotropes under both basal conditions and upon GnRH stimulation, and hence suggest that Cx36-containing gap junctions are necessary to ensure efficient, synchronous secretion of gonadotropins from the AP. It is well-documented that LH secretion varies greatly during the course of the estrous cycle, both with respect to pulse frequency and amplitude (Baird, 1978). If gap junction coupling is crucial for the generation of peak secretion as we and others argue (Guérineau et al., 1998), then Cx36 expression should change during the course of the estrous as well. Indeed, as shown in this study, Cx36 mRNA expression in the AP was increased in estrus compared to diestrus, and after ovariectomy compared to control, i.e., during two states in which LH pulse frequency is maximal and would thus require well-synchronized secretory activity (Baird, 1978; Leipheimer and Gallo, 1983). Notably, Hodson et al. showed that synchronization between lactotropes increases demand dependently in lactating females, and that the increased synchrony is also due to augmented gap junction coupling (Hodson et al., 2012). Our results suggest that in gonadotropes the regulation of gap junction coupling is at least in part supported by an increased expression of Cx36.

The increase of Cx36 expression following ovariectomy implies gonadal steroids as possible mediators (Weick and Noh, 1984). Testing this directly, we found indeed that estradiol reduced Cx36 expression in cultured pituitary cells. Furthermore, we detected putative estradiol response elements within the Cx36 promoter, which together with the fast time course of the decrease in mRNA levels, lends credence to a scenario in which estradiol is a direct regulator of Cx36 expression. The estradiol induced reduction of Cx36 mRNA expression in cell culture cannot be directly compared with the *in vivo* scenario where other estradiol kinetics may be expected. Hence, further research is warranted to understand how estradiol levels in each estrus phase translate into gap junction coupling of differential strength. Also, ovariectomy and cell culture are artificial situations that deprive gonadotropes of more than one factor. However, they prove useful in obtaining mechanistic insight as was the case in this study where we could show that estradiol is one factor (several others are likely to also contribute) that regulates the expression of Cx36 and hence the extent of gap junction communication between gonadotropes. These results suggest an additional negative estradiol-mediated feedback loop that targets the fine-tuning of LH release in the pituitary gland by reducing the synchronization of gonadotropes following phases of physiologically high estradiol.

Thus, a local mechanism of electrotonically coupled gonadotropes at the pituitary level must be added to the complex system controlling female reproduction, and alterations of the intra-pituitary crosstalk may contribute to the pathogenesis of frequently occurring disruptions of menstrual cycle, anovulation and infertility in women (Fritz and Speroff, 2010; Unuane et al., 2011; ESHRE Capri Workshop Group, 2012).

## Author contributions

CG, HM, and VG conceived the study. CG, DG, CL, LR, and DB performed experiments. CG, DG, CL, and DB analyzed data. CG, DG, CL, VG, and HM wrote the paper.

## Funding

GR 3619/2-1 and GR 3619/3-1 grants from the German Research Foundation (DFG), and grant within the Collaborative Research Center (SFB) 1134 to VG.

### Conflict of interest statement

The authors declare that the research was conducted in the absence of any commercial or financial relationships that could be construed as a potential conflict of interest.
