# Supplementary material for: Electrotonic Coupling in the Pituitary Supports the Hypothalamic-Pituitary-Gonadal Axis in a Sex Specific Manner
Source: Front Mol Neurosci. 2016 Aug 18;9:65. doi: 10.3389/fnmol.2016.00065 (PMC4988985; doi:10.3389/fnmol.2016.00065)
Supplement: Supplementary file 1 [file Image1.pdf]

Supplementary Material

Electrotonic Coupling in the Pituitary Governs the Hypothalamic-Pituitary-Gonadal Axis in a Sex Specific Manner

Christina Göngrich, Diego García-González, Corentin Le Magueresse, Lena Roth, Yasuhito Watanabe, Deborah J Burks, Valery Grinevich, Hannah Monyer*

*** Correspondence:** Hannah Monyer: h.monyer@dkfz-heidelberg.de

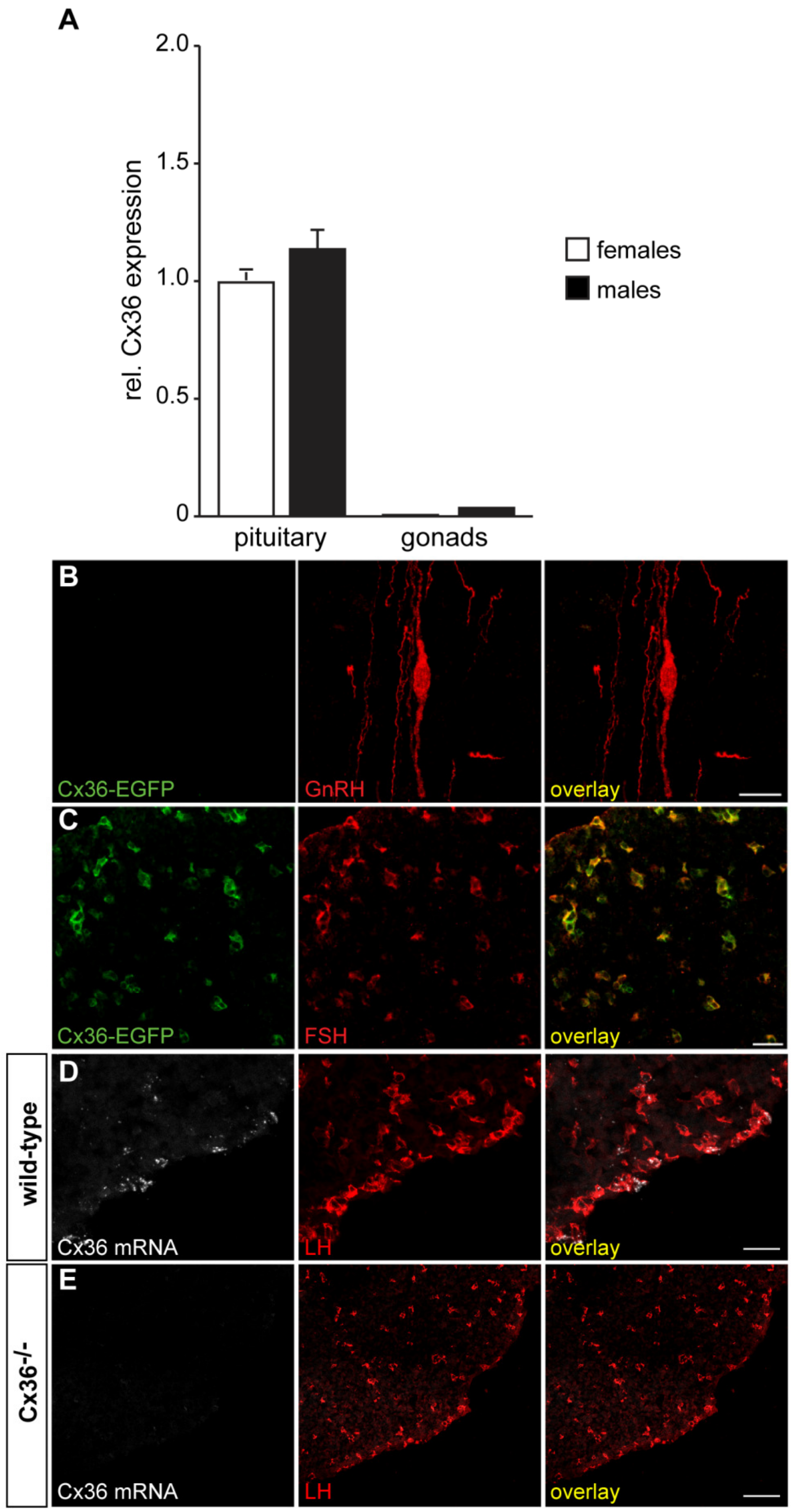

Supplementary Figure 1: FSH+ cells of the pituitary gland are coupled via Cx36-containing gap junctions.(A) Cx36 mRNA was expressed in the pituitary of female and male mice at similar levels, but was not detectable in ovaries and only at very low levels in testes of wt mice. n = 3 mice/group, mean ± SEM. (**B**) EGFP expression analysis in Cx36-EGFP mice revealed colocalization of Cx36 with FSH in the AP. Scale bar 40 µm. (C) Cx36-EGFP is not

expressed in $GnRH^+$ cells. Immunostaining showed no EGFP expression in GnRH neurons in the hypothalamus of female Cx36-EGFP mice in estrus. n = 4 mice, scale bar 40 µm. (D) Cx36 mRNA was expressed in LH-expressing gonadotrope cells in the pituitary of wild-type female mice, as revealed by double staining using *in situ* hybridization (Cx36 mRNA) and immunohistochemistry (antibody against LH). n = 1 mouse, scale bar: 40 µm. (**E**) No expression of Cx36 mRNA was found in the pituitary gland of $Cx36^{-/-}$ female mice. n = 1 mouse, scale bar: 60 µm.

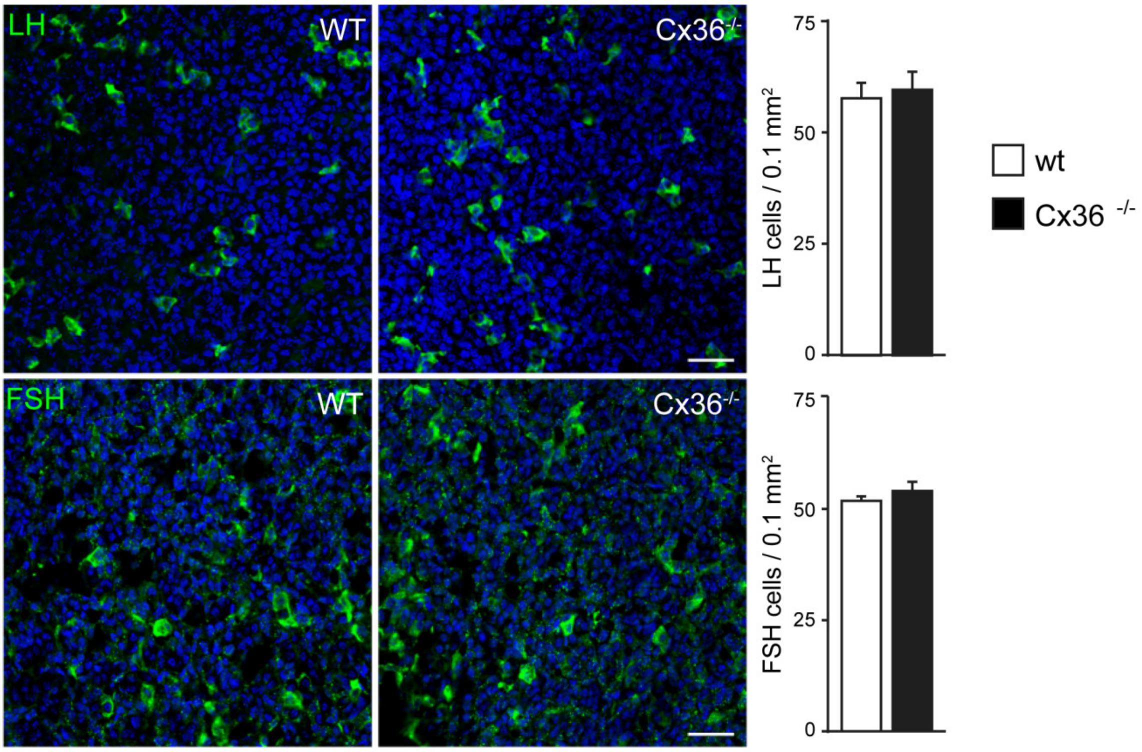

Supplementary Figure 2: The overall number of LH+ and FSH+ cells does not differ between wt and $Cx36^{-/-}$ mice. Representative confocal images of immunohistochemically labeled LH and FSH expressing cells in the AP of wt and $Cx36^{-/-}$ females. LH and FSH expressing cells were quantified using coronal pituitary sections of females in estrus. n = 3 mice for each genotype, 8-10 images from 2-3 sections/mouse were analyzed; Mean ± SEM, Student's t-test, scale bars: 40 µm.

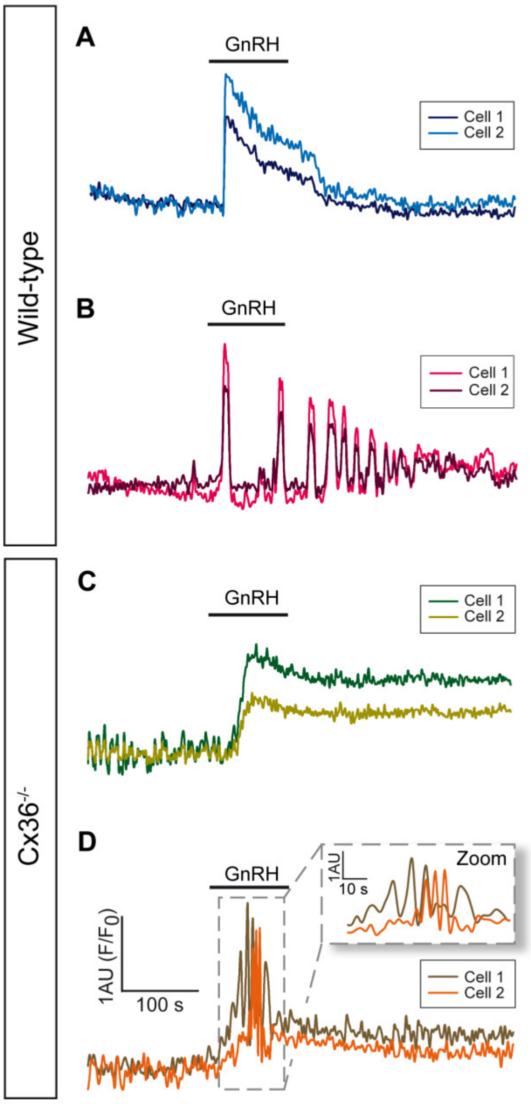

Supplementary Figure 3: GnRH induces biphasic and oscillatory Ca^{2+} responses in AP cells. Representative examples of Ca^{2+} recordings from AP slices obtained from wild-type (**A-B**) and $Cx36^{-/-}$ (**C-D**) mice. Typically, GnRH-responsive cell pairs from both genotypes exhibited two types of activity: biphasic (**A,C**) or oscillatory (**B,D**). GnRH application (1 nM) is indicated with horizontal bars.

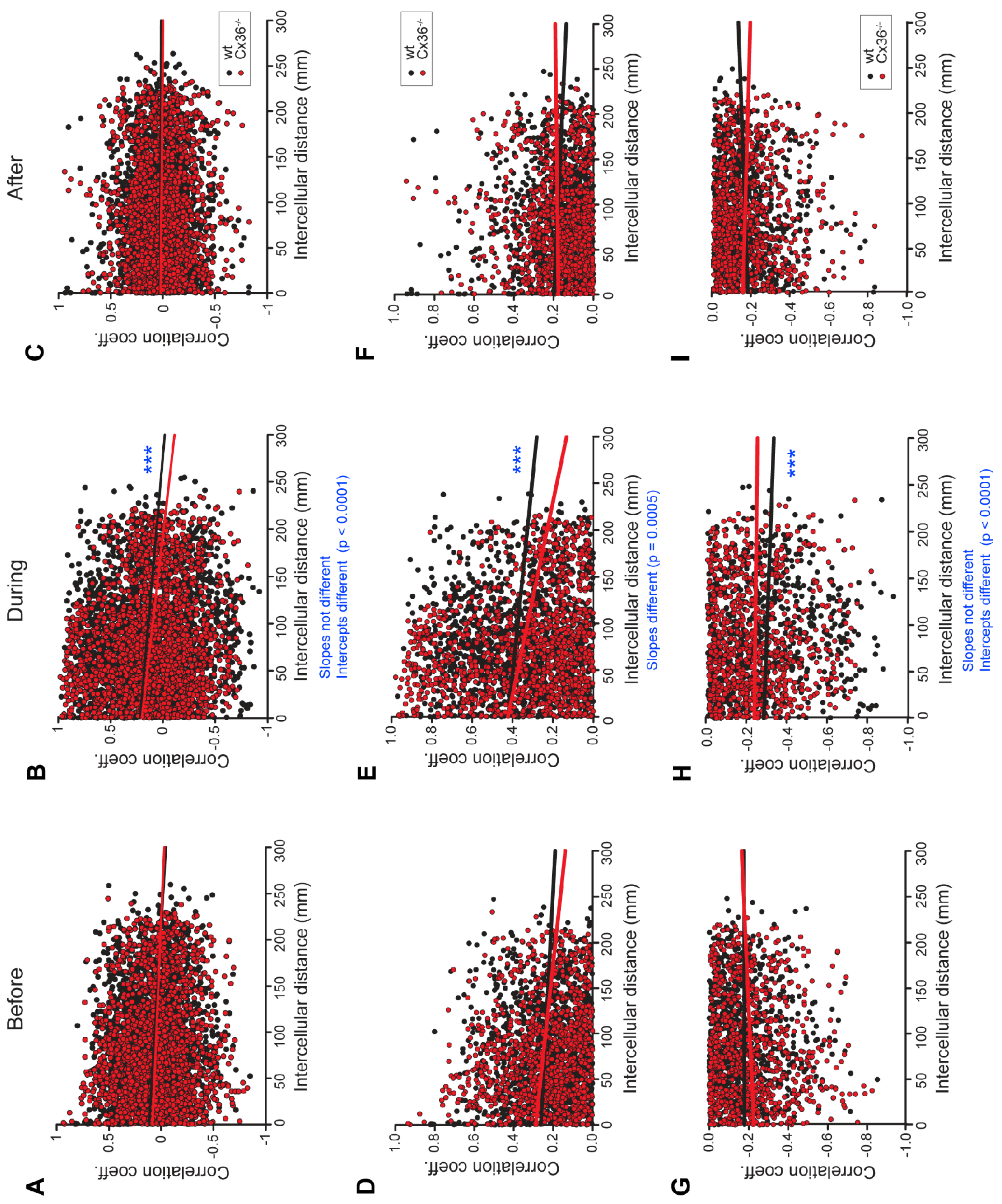

Supplementary Figure 4: Decreased synchrony between AP cell pairs in Cx36$^{-/-}$ mice during GnRH application. Spearman correlation coefficients and linear regression analysis for AP cell pairs before (**A**), during (**B**) and after (**C**) GnRH application (1 nM). In **A-C**, all correlation coefficient values are plotted on a -1 to 1 scale, i.e. synchronous calcium increase or decrease in a defined cell pair would result in a positive value, whilst anti-correlated changes would

result in a negative value. During GnRH application (**B**), regression line intercepts were significantly different ($P < 0.0001$), suggesting that Cx36 ablation induced a lower correlation degree in $Cx36^{-/-}$ mice. To further explore a possible differential effect between correlated and anti-correlated cell pairs, plots were subdivided into positive and negative Spearman correlation coefficients. In **D-F** only positive correlation coefficients were considered. During GnRH application (**E**), linear regression slopes were significantly different ($P = 0.0005$) between both groups. Similarly, in **G-I** only negative correlation coefficients were selected. In **H**, although regression slopes did not differ, intercepts were different between wt and $Cx36^{-/-}$ cells ($P < 0.0001$). Correlation coefficients between cells from wt mice reached more positive and negative values compared to $Cx36^{-/-}$ in the analysis shown in **E** and **H** respectively. This indicates a higher degree of correlated activity in wt cells with values closer to 1 and -1. During GnRH application, these opposed tendencies to extreme values in **E** and **H** partially masked the global effect in **B**, when the whole population of cell pairs was analyzed. After GnRH exposure (**C, F** and **I**), no correlation was observed between correlation coefficients and intercellular distances in wt and $Cx36^{-/-}$ cells. Number of cell pairs: n (wt) = 1908; n ($Cx36^{-/-}$) = 1951.

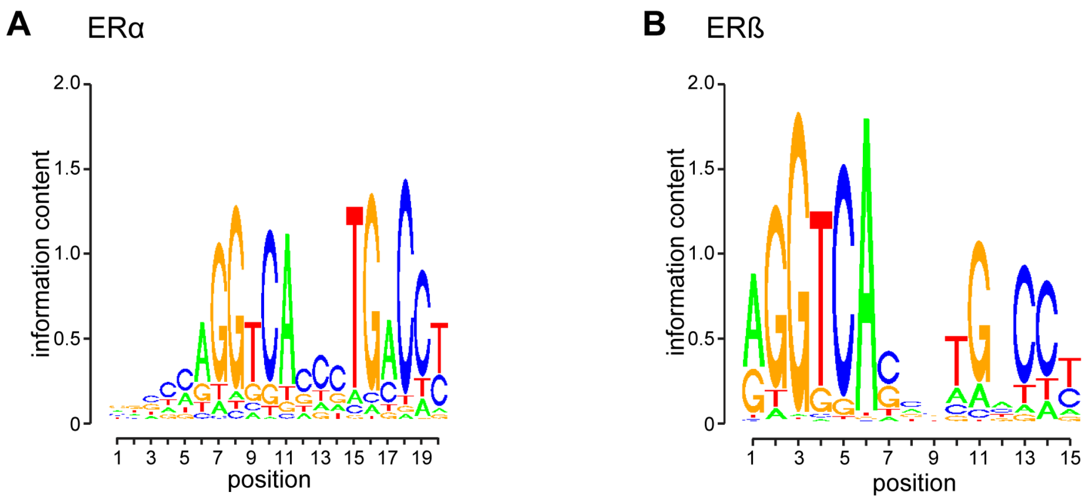

-725bp

5'-AGG**C**TG**G**AC**CA**TGC**TGACCT**-3'

-3109bp

5'-TT**CCCAGGT**GC**C**TA**TG**T**CCT**-3'

-2458bp

5'-**AGGTCA**GAGA**G**G**CC**C-3'

Supplementary Figure 5: The Cx36 promoter contains putative binding sites for ERα and ERß. Sequence Logos showing the consensus sequence for the ERα (**A**) and ERß (**B**) binding sites. Sequence analysis of 5kb upstream of the transcription start site of Cx36 revealed two putative binding sites for ERα and one for ERß. ERα sites were found at position -725 bp and at position -3109 bp (both 75 % score). The putative ERß binding site was located at position -2458 bp (88 % score). The sequences are depicted below the respective sequence logos, conserved residues are shown in bold typeface.
